# Mendelian randomization analysis of the causal relationship between COPD and type 1 and type 2 diabetes mellitus

**DOI:** 10.1097/MD.0000000000045935

**Published:** 2025-11-28

**Authors:** You Wu, You Zhou, Yueshan Pang, Lisha Zhu

**Affiliations:** a Department of Gerontology, Beijing Anzhen Nanchong Hospital of Capital Medical University & Nanchong Central Hospital, Nanchong, Sichuan, China; b The Second Clinical Medical College of North Sichuan Medical College, Nanchong, Sichuan, China; c Intensive Care Unit, Beijing Anzhen Nanchong Hospital of Capital Medical University & Nanchong Central Hospital, Nanchong, Sichuan, China; d Department of Pharmacy, Beijing Anzhen Nanchong Hospital of Capital Medical University & Nanchong Central Hospital, Nanchong, Sichuan, China.

**Keywords:** chronic obstructive, diabetes mellitus, Mendelian randomization analysis, pulmonary disease

## Abstract

This study investigated the potential causal relationship between chronic obstructive pulmonary disease (COPD) and diabetes mellitus in European and East Asian populations using Mendelian randomization (MR) analysis. Single nucleotide polymorphisms associated with COPD, type 1 diabetes mellitus (T1DM), and type 2 diabetes mellitus (T2DM) were obtained from large-scale genome-wide association studies and used as instrumental variables. Three MR methods were applied: inverse variance weighting, MR-Egger regression, and the weighted median approach. Our analysis found a marginal causal association between genetically predicted T1DM and an increased risk of COPD in the European population (odds ratio = 1.9109, 95% confidence interval 1.0003–3.0016, *P* = .005). However, no significant causal relationship was observed between COPD and T2DM in either direction. Sensitivity analyses suggested no evidence of directional pleiotropy or heterogeneity, thus supporting the robustness of the findings. Nonetheless, the *P*-value for the simple mode method was above .05, indicating some uncertainty in the results. While a potential causal effect of T1DM on COPD in European populations, the weak statistical signals and marginal odds ratios warrant caution in interpreting these findings. Furthermore, no causal link between COPD and T2DM was observed. The study’s findings should be considered preliminary, and further research with larger, more diverse cohorts and additional mechanistic insights are necessary to validate and extend these results.

**Key points**

- *Type 1 diabetes mellitus (T1DM) as a COPD risk factor*: This study establishes a causal link between T1DM and chronic obstructive pulmonary disease (COPD) in Europeans, providing new insights into T1DM’s role in COPD.
- *No link between COPD and type 2 diabetes mellitus (T2DM*): The study shows no causal relationship between COPD and T2DM, clarifying the COPD–diabetes connection.
- *Supporting literature*: Reinforces the association between diabetes and COPD, particularly T1DM, as a risk factor.
- *Clinical implications*: Highlights the need for COPD monitoring in patients with T1DM and suggests early intervention strategies.

## 1. Introduction

Chronic obstructive pulmonary disease (COPD) is one of the most prevalent lung diseases worldwide, and poses a significant public health challenge. According to the 2021 Global Burden of Disease Study, COPD has become the third leading cause of age-standardized mortality globally, severely threatening long-term quality of life and imposing a heavy social and economic burden.^[1,2]^ In 2017, approximately 544.9 million people worldwide were affected by chronic respiratory diseases, and it is projected that by 2030, COPD will be the third leading cause of death globally.^[3]^

Diabetes mellitus (DM) is also a common disease worldwide,^[4]^ with an estimated 366 million people living with diabetes in 2011, which is expected to increase to 552 million by 2030.^[5]^ Growing evidence suggests that COPD is associated with a range of comorbidities including neurodegenerative diseases, cardiovascular events, osteoporosis, malignancies, and mortality.^[6,7]^ Current evidence also indicates that COPD and diabetes may be linked through common pathophysiological mechanisms involving shared risk factors such as chronic inflammation, oxidative stress, and reduced physical activity. Additionally, COPD treatment, particularly the use of corticosteroids, may exacerbate blood glucose metabolism abnormalities, further complicating the management of diabetes.^[8]^ However, there is currently no clear evidence of a direct causal relationship between COPD and diabetes.

While observational studies have reported higher rates of diabetes among COPD patients, these studies are limited by confounding factors, reverse causality, and other biases inherent in traditional research designs. For example, a population-based retrospective study from Italy found that the prevalence of DM among COPD patients (18.7%) was higher than that in the general population (10.5%), and women with COPD had a significantly higher risk of developing type 2 diabetes mellitus (T2DM) than those without COPD.^[9]^ A large study involving 341,329 Italian participants found a lower prevalence of diabetes in individuals without COPD compared to those with the disease.^[10]^ Currently, while there is growing observational evidence linking COPD with diabetes, few studies have rigorously addressed the causal nature of this relationship. Moreover, the specific causal roles of different diabetes subtypes, particularly type 1 diabetes mellitus (T1DM) and T2DM, in COPD development remain underexplored.

This gap in understanding the causal relationship between COPD and diabetes has been highlighted in recent systematic reviews, which have pointed out the need for more robust studies to clarify the direction and nature of this association.^[11]^ Confounding factors, such as age, smoking history, and socioeconomic status, complicate causal inferences, and reverse causality further muddles the understanding of how these diseases interact. Despite the prevalence of observational studies, causal inference has remained elusive, making it difficult to establish a clear, direct link between COPD and diabetes.

Traditional observational studies are inherently limited in establishing causality due to residual confounding and reverse causality. Mendelian randomization (MR), which uses genetic variants as instrumental variables (IVs) to infer causal relationships, offers a promising alternative to minimize the impact of such confounders. Although MR has been widely applied to explore causal links between various diseases, studies investigating the causal relationship between COPD and diabetes, particularly distinguishing between T1DM and T2DM, remain scarce and are underrepresented in the current literature.

Therefore, this study seeks to address these critical gaps by employing a 2-sample MR analysis to explore the bidirectional causal relationship between COPD and both T1DM and T2DM. By leveraging large-scale genome-wide association study (GWAS) data from European and East Asian populations, our study aims to clarify whether diabetes causally increases the risk of COPD or whether COPD predisposes individuals to diabetes. Understanding these causal pathways could have important implications for early identification, prevention, and integrated management strategies for individuals at risk of both COPD and diabetes.

## 2. Materials and methods

### 2.1. Study design

**Figure 1.**
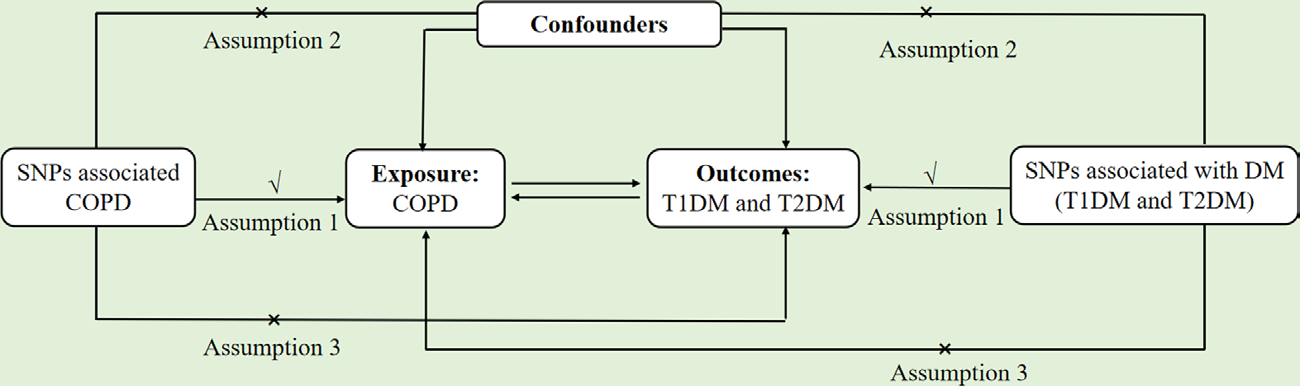
Flowchart of the data collection, processing, and analysis procedures of this study.

This study uses single nucleotide polymorphisms (SNPs) associated with COPD and both T1DM and T2DM as IVs to explore the potential causal relationships between COPD and T1DM, as well as between COPD and T2DM. MR analysis relies on 3 core assumptions^[12]^: genetic variations are strongly associated with exposure; genetic variations are not related to confounding factors; and genetic variations influence the outcome solely through exposure (Fig. 1). We conducted MR analysis using data from published GWAS. As each study obtained approval from its respective ethics committee, no further ethical approval was required.

### 2.2. Instrumental variables selection

The GWAS summary data used in this study were sourced from the Integrative Epidemiology Unit GWAS database (https://gwas.mrcieu.ac.uk/). GWAS data for COPD and T2DM from European and East Asian populations, as well as European population data for T1DM, were included (see Table 1). SNPs with genome-wide significance were selected from the GWAS data. The *P*-value threshold for initial screening was set to ensure strong associations with the exposure variable for each dataset, as detailed in Table 1. To minimize the impact of linkage disequilibrium on the analysis, an *R*² threshold of 0.001 and window size of 10,000 kb were set. Additionally, SNPs associated with other phenotypes were excluded using PhenoScanner (http://www.phenoscanner.medschl.cam.ac.uk/). Furthermore, an *F*-statistic >10 indicated a lower probability of bias due to weak instrument variables and was used to assess the presence of weak instrument bias for the selected SNPs (see Table S1, Supplemental Digital Content, https://links.lww.com/MD/Q619).

**Table 1:** Detailed information for the GWAS data.

Diseases	GWAS ID	Data source	Population	Sample size (cases/controls)	Number of SNPs	Years	*P* val
COPD	ukb-b-16751	IEU GWAS database	European	3871/459,139	9,851,867	2018	*P* < 5e−5
COPD	bbj-a-103	IEU GWAS database	East Asian	3315/201,592	8,885,538	2019	*P* < 5e−6
T1DM	ebi-a-GCST005536	IEU GWAS database	European	6683/12,173	101,101	2015	*P* < 5e−10
T2DM	ukb-b-13806	IEU GWAS database	European	2972/459,961	9,851,867	2018	*P* < 5e−7
T2DM	ebi-a-GCST010118	IEU GWAS database	East Asian	77,418/356,122	11,222,507	2020	*P* < 5e−20

COPD = chronic obstructive pulmonary disease, GWAS = genome-wide association study, IEU = Integrative Epidemiology Unit, SNP = single nucleotide polymorphism, T1DM = type 1 diabetes mellitus, T2DM = type 2 diabetes mellitus.

### 2.3. Data sources

Table 1 summarizes the characteristics of the GWAS datasets used in this study. The summary GWAS data used in this study was obtained from the Integrative Epidemiology Unit OpenGWAS project (https://gwas.mrcieu.ac.uk/datasets/). The genetic background of the MR study population was limited to individuals of European and East Asian ancestries. The data used in this study are publicly available, and each original study received ethical approval and informed consent.

### 2.4. MR and statistical analysis

This study used R software (version 4.4.0; R Foundation for Statistical Computing, Vienna, Austria) and the “TwoSampleMR” package (R Core Team, Vienna, Austria) to conduct 2-sample MR analysis. The primary method employed to estimate the causal relationship between COPD and diabetes is inverse variance weighting (IVW), as this method provides accurate causal effect estimates under the assumption of valid IVs.^[13,14]^ To assess the robustness of the results, several additional analyses were conducted, including the MR-Egger regression, weighted median, and weighted mode methods. These methods account for different types of genetic pleiotropy and are based on varying assumptions.^[14–16]^ In contrast to the IVW method, MR-Egger regression assesses the presence of pleiotropy in the IVs through the regression intercept, although its efficiency is generally lower compared to other methods, particularly when pleiotropy is present.^[17]^ When the proportion of invalid instruments reaches 50%, the weighted median method can provide correct causal effect estimates.^[18]^ In addition, the weighted mode method assumes that the most common causal estimate is not influenced by pleiotropy, thereby providing a single causal effect estimate.^[19]^

To test for heterogeneity among the IVs, Cochran *Q* statistic was calculated using both IVW and MR-Egger regression. A *P*-value >.05 indicates no significant heterogeneity among the instruments.^[20]^ Furthermore, the Mendelian Randomization Pleiotropy RESidual Sum and Outlier (MR-PRESSO) test was used to detect potential pleiotropy and to exclude outliers. The intercept term in the MR-Egger regression was used to examine the horizontal pleiotropic bias of the included SNPs; an intercept close to zero suggests no horizontal pleiotropy of the IVs.^[16]^ Finally, a sensitivity analysis was performed using leave-one-out analysis to assess the impact of removing a single SNP on causal effect estimates. A *P*-value >.05 after removing an SNP indicates that the SNP does not significantly affect the results.^[21]^ MR results are presented as odds ratios (OR) with 95% confidence intervals, with the significance level set at α = 0.05 (2-sided). Sample size calculations for statistical power were conducted using a priori methods, and the effect sizes for the SNPs used in the analyses were assessed to ensure that the study was sufficiently powered to detect meaningful causal relationships. This helps justify the use of the selected SNPs and ensures robust results.

### 2.5. Ethical approval

Research not involving humans does not require ethical approval in accordance with local legislation and institutional requirements.

## 3. Results

### 3.1. COPD and DM in European population

**Figure 2.**
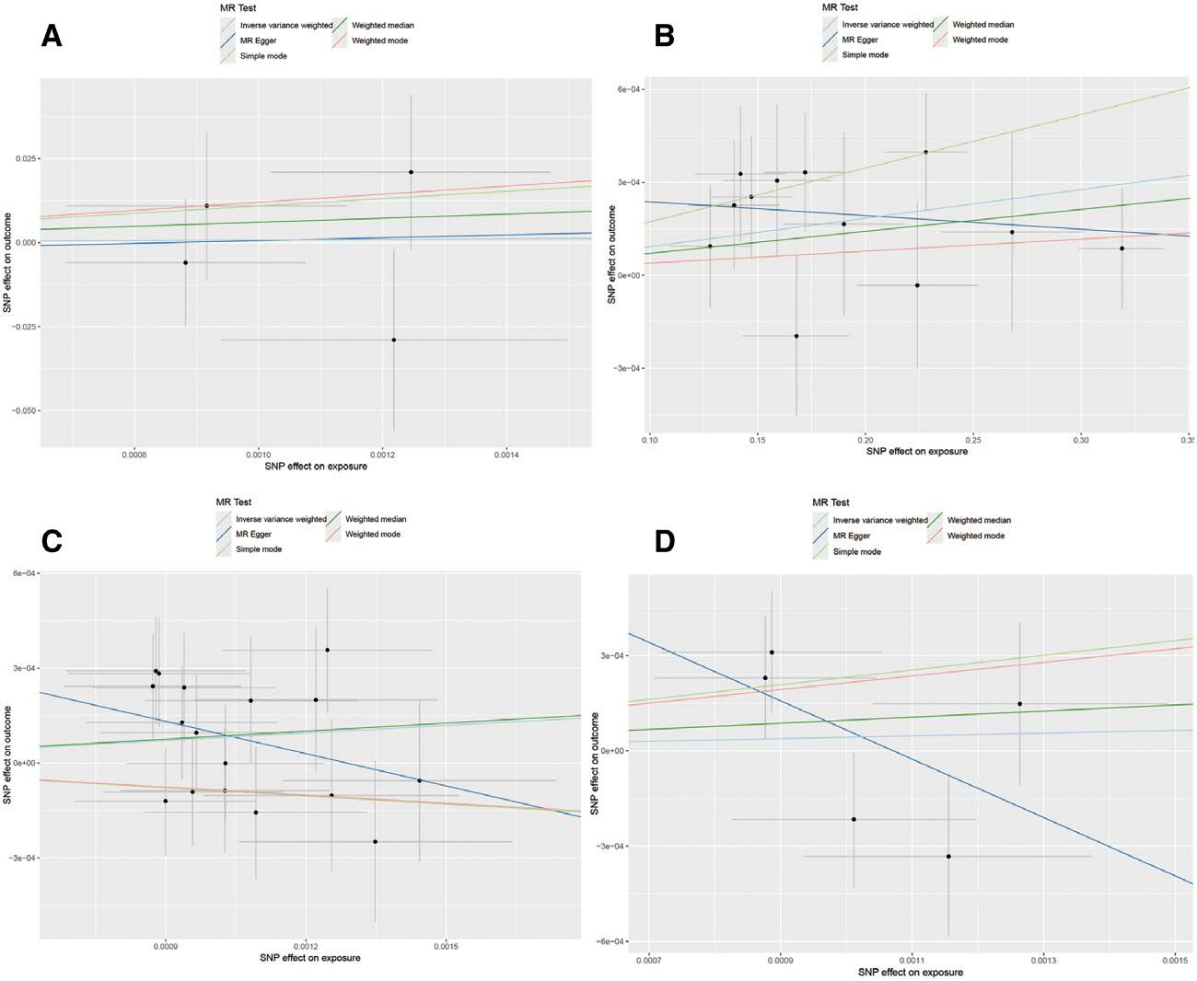
Scatter plots for European population: forward MR analyses (A: COPD–T1DM; C: COPD–T2DM) and reverse MR analyses (B: T1DM–COPD; D: T2DM–COPD). COPD = chronic obstructive pulmonary disease, MR = Mendelian randomization, T1DM = type 1 diabetes mellitus, T2DM = type 2 diabetes mellitus.

IVW analysis showed no significant causal association between COPD and T1DM or T2DM in the European population. Additionally, the results of MR-Egger regression and weighted median methods also failed to identify any genetic causal relationship between COPD and either T1DM or T2DM (Table 2, Fig. 2A and C). The results of the Cochran *Q* test, MR-PRESSO, and MR-Egger tests are presented in Table 3. According to the Cochran *Q* test, no significant heterogeneity was observed between SNPs. Therefore, causal inference in this study was primarily based on the fixed-effect IVW model. The MR-PRESSO analysis revealed no evidence of pleiotropy or outliers. The MR-Egger test results indicated no significant horizontal pleiotropy. The funnel plot and leave-one-out sensitivity analysis for COPD and diabetes showed that the removal of any individual SNP did not significantly affect the robustness of the results (Fig. S1A–D, Supplemental Digital Content, https://links.lww.com/MD/Q619).

**Table 2:** MR results between COPD and DM.

Exposure	Outcome	Population	Method	nSNP	*P* value	OR	LCI	UCI
*The forward MR analyses*								
COPD	T1DM	European	MR Egger	4	.959	0.9823	−0.9490	1.3579
			Weighted median	4	.638	3.1221	0.3928	5.5348
			Inverse variance weighted	4	.939	2.2567	−1.9695	2.5892
			Simple mode	4	.624	1.4434	−2.2259	4.0600
			Weighted mode	4	.535	1.4500	−2.0926	3.9234
COPD	T2DM	European	MR Egger	17	.279	0.7118	0.3931	1.2888
			Weighted median	17	.176	1.0876	0.9631	1.2283
			Inverse variance weighted	17	.078	1.0823	0.9912	1.1819
			Simple mode	17	.510	0.9169	0.7122	1.1804
			Weighted mode	17	.519	0.9188	0.7143	1.1818
COPD	T2DM	East Asian	MR Egger	7	.491	0.7913	0.4267	1.4673
			Weighted median	7	.266	0.9675	0.9127	1.0255
			Inverse variance weighted	7	.190	0.9462	0.8709	1.0279
			Simple mode	7	.630	0.9620	0.8285	1.1171
			Weighted mode	7	.886	0.9908	0.8772	1.1191
*The reverse MR analyses*								
T1DM	COPD	European	MR Egger	12	.695	0.9996	0.9974	1.0017
			Weighted median	12	.095	1.1007	0.2999	1.5015
			Inverse variance weighted	12	**.005**	1.9109	1.0003	3.0016
			Simple mode	12	**.045**	1.0017	0.0002	2.0033
			Weighted mode	12	.541	1.0004	0.9992	1.0016
T2DM	COPD	European	MR Egger	5	.364	0.3989	0.0739	2.1540
			Weighted median	5	.484	1.1011	0.8405	1.4425
			Inverse variance weighted	5	.733	1.0437	0.8159	1.3351
			Simple mode	5	.353	1.2603	0.8183	1.9410
			Weighted mode	5	.366	1.2389	0.8202	1.8713
T2DM	COPD	East Asian	MR Egger	47	.909	1.0186	0.7445	1.3937
			Weighted median	47	.288	0.9223	0.7944	1.0707
			Inverse variance weighted	47	.067	0.9067	0.8165	1.0069
			Simple mode	47	.961	0.9920	0.7187	1.3693
			Weighted mode	47	.879	1.0212	0.7811	1.3351

Bold values indicate statistical significance (*P* < .05).

COPD = chronic obstructive pulmonary disease, MR = Mendelian randomization, OR = odds ratio, SNP = single nucleotide polymorphism, T1DM = type 1 diabetes mellitus, T2DM = type 2 diabetes mellitus.

**Table 3:** Pleiotropy and heterogeneity analyses for the association of COPD and DM.

Exposure	Outcome	Population	Disease	Heterogeneity test						Pleiotropy test		
			No. of SNPs	MR-Egger			IVW					
				Q	Q_df	Q_pval	Q	Q_df	Q_pval	Egger_	SE	*P*-val
										Intercept		
*The forward MR analyses*												
COPD	T1DM	European	4	2.329	2	0.312	2.331	3	0.507	−0.004	0.075	.966
COPD	T2DM	European	17	13.260	15	0.5822	15.218	16	0.509	0.000	0.000	.182
COPD	T2DM	East Asian	7	34.110	5	0.226	36.351	6	0.236	0.030	0.052	.591
*The reverse MR analyses*												
T1DM	COPD	European	12	5.723	10	0.838	7.423	11	0.764	0.001	0.001	.222
T2DM	COPD	European	5	4.913	3	0.178	7.000	4	0.136	0.000	0.001	.341
T2DM	COPD	East Asian	47	52.653	45	0.202	53.351	46	0.213	−0.011	0.014	.444

COPD = chronic obstructive pulmonary disease, IVW = inverse variance weighting, MR = Mendelian randomization, SNP = single nucleotide polymorphism, T1DM = type 1 diabetes mellitus, T2DM = type 2 diabetes mellitus.

In the reverse Mendelian randomization analysis, we found a causal relationship between T1DM and COPD in a European population. The IVW analysis showed an OR = 1.9109, 95% confidence interval: 1.0003–3.0016, *P* = .005. This result was also supported by the simple mode method (Table 2, Fig. 2B). However, no causal relationship was observed between T2DM and COPD, and the results of the other MR analyses were consistent with the IVW findings (Table 2, Fig. 2D). Both the MR-Egger regression and MR-PRESSO results showed no evidence of potential pleiotropy (Table 3). Cochran *Q* test in both the IVW and MR-Egger methods indicated no significant heterogeneity between IVs (Table 3). Furthermore, leave-one-out analysis showed that the observed associations did not significantly change after the removal of any individual SNP (Fig. S2A–D, Supplemental Digital Content, https://links.lww.com/MD/Q619), further confirming the stability of the results.

### 3.2. COPD and DM in East Asian population

**Figure 3.**
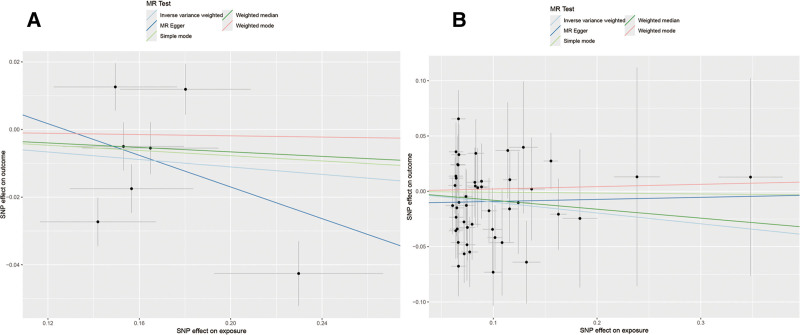
Scatter plots for East Asian population: forward MR analyses (A: COPD–T2DM) and reverse MR analyses (B: T2DM–COPD). MR = Mendelian randomization, T2DM = type 2 diabetes mellitus.

The study included 7 SNPs as IVs for COPD and 47 SNPs for T2DM in the East Asian population. Both forward and reverse MR analyses using the fixed-effects IVW model found no causal relationship between COPD and diabetes in the East Asian population. This result is consistent with the findings from the MR-Egger regression and the weighted median method (Table 2, Fig. 3A and B). Cochran *Q* test indicated no significant heterogeneity among the IVs. Additionally, MR-Egger and MR-PRESSO regressions did not detect significant horizontal pleiotropy (Table 3). The funnel plots and leave-one-out sensitivity analyses for COPD and diabetes in the East Asian population are shown in Figs. S1E, F and S2E, F, Supplemental Digital Content, https://links.lww.com/MD/Q619. Leave-one-out analysis indicated that the statistical results remained stable, with no significant changes after excluding any single SNP.

### 3.3. Enrichment analyses

**Figure 4.**
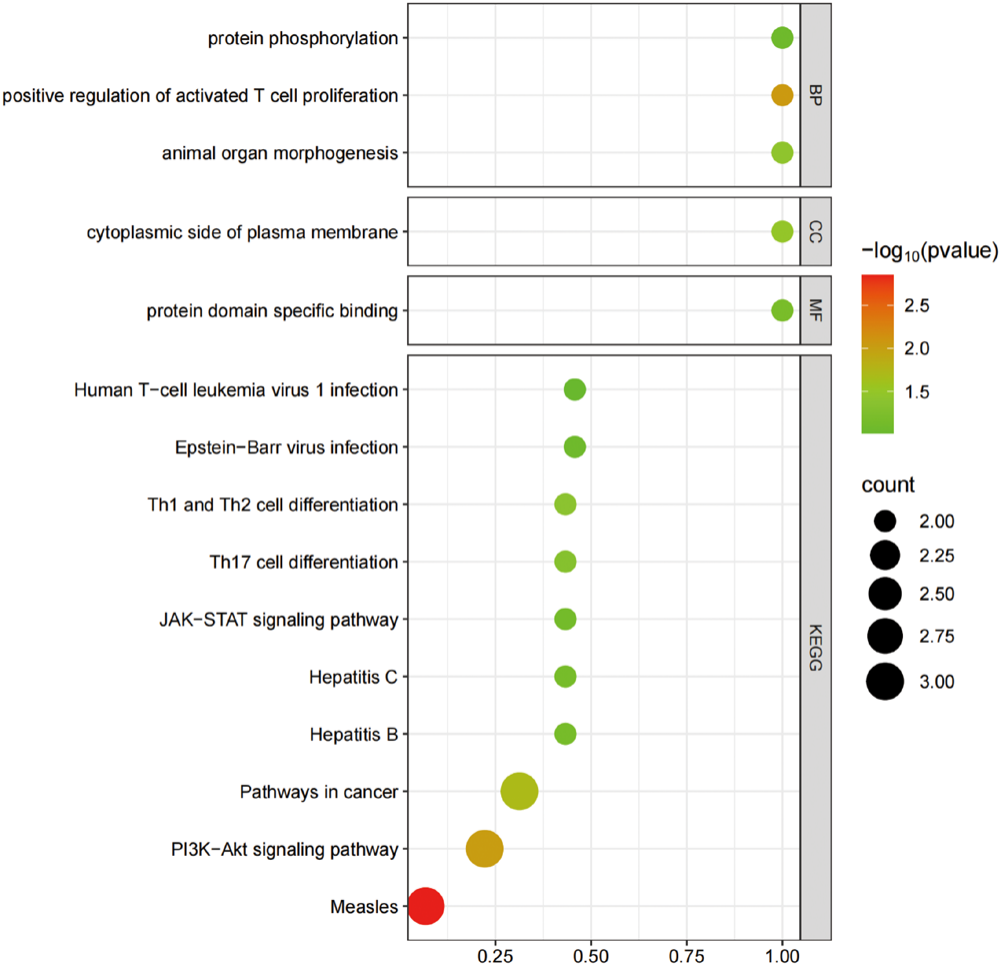
A bubble chart of GO and KEGG enrichment analysis. GO = gene ontology, KEGG = Kyoto Encyclopedia of Genes and Genomes.

We performed gene ontology and Kyoto Encyclopedia of Genes and Genomes (KEGG) enrichment analyses of SNPs inversely associated with COPD and T1DM, with the corresponding gene names provided in Table S2, Supplemental Digital Content, https://links.lww.com/MD/Q619. Gene ontologyanalysis indicated that protein phosphorylation, positive regulation of activated T cell proliferation, and animal organ morphogenesis are linked to biological processes, the cytoplasmic side of the plasma membrane is associated with cellular components, and protein domain-specific binding pertains to molecular functions. KEGG analysis further highlighted significantly enriched pathways, including measles, PI3K-Akt signaling pathway, cancer-related pathways, Th1 and Th2 cell differentiation, and Th17 cell differentiation (Fig. 4).

## 4. Discussion

This study employed a bidirectional MR analysis to investigate whether genetic evidence supports a causal relationship between COPD and DM. Understanding the role of COPD in diabetes could provide insight into its contribution to the onset and progression of the disease. In the reverse Mendelian randomization analysis, we found evidence of a causal relationship between T1DM and COPD in a European population. This finding suggests that COPD may play a role in the development and progression of T1DM. T1DM, an autoimmune disorder, can aggravate immune system dysregulation in COPD patients, potentially leading to disease progression and worsening.^[22]^ In genetically susceptible individuals, COPD may worsen immune responses, further accelerating the onset or progression of T1DM.^[23]^ However, given the weak effect size and statistical significance (OR near 1.0009), we recognize that this association may not be biologically relevant enough to be considered clinically actionable. Further studies with larger sample sizes and stronger statistical power are needed to validate this finding and explore its practical implications.

Previous studies have indicated a potential link between COPD and immune dysfunction, inflammatory response, and metabolic disorders. This is consistent with the pathways identified in our KEGG enrichment analysis, particularly the PI3K-Akt signaling pathway. These mechanisms may provide insights into the causal relationship between type 1 diabetes and COPD.^[8,11,24]^ This pathway plays a dual role in both conditions: in T1DM, it regulates β-cell survival and T cell-mediated islet destruction, whereas in COPD, it contributes to chronic airway inflammation and corticosteroid resistance under oxidative stress.^[25]^ While these findings offer some biological plausibility for the association between T1DM and COPD, we emphasize that the current evidence is far from conclusive, and this hypothesis remains to be tested through experimental and longitudinal studies.

However, in both the forward and reverse MR analyses conducted in European and East Asian populations, we did not find a significant causal relationship between COPD and T2DM. Clinical studies investigating the relationship between COPD and T2DM are limited, and have yielded inconsistent results. For instance, some studies have suggested an increased risk of COPD in T2DM patients,^[26,27]^ while a retrospective case–control study of 29,217 diabetic patients over 8 years found a decreased risk of COPD in T2DM patients.^[28]^ Notably, our findings are consistent with those of other studies. For example, a study of 3466 T2DM patients aged 40 to 80 years in Korea found a significant association between restrictive ventilatory defects and T2DM but no significant association between COPD and T2DM.^[29]^ Similarly, a 5-year prospective study in Japan, including 7524 participants aged 40 to 69 with no baseline lung function impairment, found a significant association between diabetes and restrictive lung dysfunction but no significant association with COPD.^[30]^ These results are in line with those of our study, suggesting no significant relationship between COPD and increased risk of T2DM.

This lack of a causal relationship may be attributed to the complex etiology of T2DM and potential differences in the mechanisms underlying COPD. T2DM is typically closely associated with lifestyle, obesity, diet, and other environmental factors with a more complex genetic basis, whereas COPD is primarily caused by long-term smoking and other environmental factors, which could lead to a weaker direct genetic link between the 2.^[31–33]^ While COPD may contribute to metabolic disturbances in T2DM patients, its role in the causal pathway seems more limited.

It is crucial to note that although our MR analysis did not reveal a significant causal relationship between COPD and T2DM, this does not rule out potential indirect associations. COPD may play an indirect role in certain complications of T2DM, such as cardiovascular disease or renal dysfunction.^[34,35]^ Given the complexity of T2DM and its interactions with other conditions, future research should explore how COPD influences various aspects of T2DM, particularly in the development of chronic complications.

The immune dysregulation observed in COPD may also accelerate the onset or progression of T1DM in genetically predisposed individuals. Our KEGG enrichment analysis identified immune-related pathways, such as PI3K-Akt signaling, Th1/Th2/Th17 cell differentiation, and cytokine–cytokine receptor interactions, which are central to both innate and adaptive immunity. Cigarette smoke exposure activates the PI3K pathway, leading to IL-17A/F upregulation in lung tissue.^[36]^ This may link airway inflammation to systemic immune dysregulation. Furthermore, the Th17/Treg imbalance, commonly seen in both COPD and metabolic diseases, could represent a shared immune disturbance that promotes chronic inflammation.^[37,38]^ In T1DM, Th17, and IL-17 contribute to β-cell autoimmunity, with IL-17 blockade showing protective effects in preclinical models.^[39]^ These immune-related mechanisms warrant further investigation in experimental studies.

This study applied bidirectional MR analysis, a method that effectively addresses common confounding factors and reverse causality issues in observational studies using genetic variants as IVs, thus providing more reliable causal inferences. This study utilized large-scale population data covering both European and East Asian populations, enabling cross-population assessment of the causal relationship between COPD and diabetes, thereby enhancing the generalizability of the findings. Multiple analytical methods (e.g., MR-Egger regression and the weighted median method) were also employed to further validate the robustness of the results and ensure the reliability of causal inferences.

While this study applied bidirectional MR analysis to address common confounding factors and reverse causality issues, several limitations should be acknowledged. First, the statistical power of the analysis was limited, especially for the COPD–T1DM relationship due to the small number of SNPs available (4 SNPs). Although all selected SNPs met the *F*-statistic threshold for strong IVs, the statistical precision of our findings could be improved with more SNPs or larger datasets. Second, the possibility of population stratification bias remains, as subtle genetic differences between European and East Asian cohorts may still confound the causal estimates. Third, while genetic instruments are less prone to confounding by environmental factors, unmeasured confounders such as socioeconomic status, diet, and healthcare access may still influence the results. Finally, the cross-sectional nature of the underlying GWAS data limits our ability to draw conclusions about temporal dynamics between COPD and diabetes.

In conclusion, while our study offers some insight into the potential genetic relationship between COPD and diabetes, further research with larger, more diverse populations and updated longitudinal data is needed to clarify these associations. We hope that future studies will explore the mechanisms underlying these relationships, particularly in the context of chronic complications in T2DM patients and immune dysregulation in COPD patients.

## Acknowledgments

All the datasets utilized in the present investigation were sourced from publicly accessible databases and collaborative consortia. The authors extend their gratitude to these entities for facilitating access to the data, which was instrumental in the execution of this research.

## Author contributions

**Conceptualization:** Lisha Zhu.

**Data curation:** You Wu, You Zhou, Yueshan Pang.

**Project administration:** Lisha Zhu.

**Visualization:** You Wu, You Zhou, Yueshan Pang.

**Writing – original draft:** You Wu.

**Writing – review & editing:** Lisha Zhu.

## Supplementary Material


