# Supplementary material for: Mendelian randomization analysis of the causal relationship between COPD and type 1 and type 2 diabetes mellitus
Source: Medicine (Baltimore). 2025 Nov 28;104(48):e45935. doi: 10.1097/MD.0000000000045935 (PMC12662473; doi:10.1097/MD.0000000000045935)

**Mendelian Randomization Analysis of the Causal Relationship Between Chronic Obstructive Pulmonary Disease and Type 1 and Type 2 Diabetes Mellitus**

Table S1. The detailed information of the instrumental variables in each trait.

| **Exposure** | **Outcome** | **Population** | SNP | Effect allele | Other allele | EAF | Beta | SE | *P* value |
| --- | --- | --- | --- | --- | --- | --- | --- | --- | --- |
| COPD | T1DM | European | rs12203592 | T | C | 0.2196 | 0.0012 | 0.0250 | 3.10E-08 |
| COPD | T1DM | European | rs62057232 | C | T | 0.3908 | -0.0009 | 0.0200 | 5.30E-06 |
| COPD | T1DM | European | rs7583953 | A | G | 0.2275 | -0.0009 | 0.0280 | 4.90E-05 |
| COPD | T1DM | European | rs9315903 | A | G | 0.1405 | -0.0012 | 0.0210 | 1.20E-05 |
| COPD | T2DM | European | rs1032297 | G | A | 0.4034 | -0.0010 | 0.0002 | 7.50E-07 |
| COPD | T2DM | European | rs10774818 | C | T | 0.7663 | -0.0010 | 0.0002 | 4.50E-06 |
| COPD | T2DM | European | rs11249992 | A | C | 0.3903 | 0.0009 | 0.0002 | 1.50E-06 |
| COPD | T2DM | European | rs11961507 | C | T | 0.1263 | -0.0013 | 0.0003 | 3.70E-06 |
| COPD | T2DM | European | rs12203592 | T | C | 0.2196 | 0.0012 | 0.0002 | 3.10E-08 |
| COPD | T2DM | European | rs12693902 | A | G | 0.6802 | 0.0010 | 0.0002 | 2.90E-06 |
| COPD | T2DM | European | rs34517439 | A | C | 0.1218 | 0.0014 | 0.0003 | 8.20E-07 |
| COPD | T2DM | European | rs3948698 | A | G | 0.4113 | -0.0009 | 0.0002 | 4.90E-06 |
| COPD | T2DM | European | rs4403440 | A | G | 0.4995 | -0.0009 | 0.0002 | 4.20E-06 |
| COPD | T2DM | European | rs56413025 | A | G | 0.2725 | 0.0010 | 0.0002 | 1.30E-06 |
| COPD | T2DM | European | rs7159635 | T | C | 0.3421 | 0.0009 | 0.0002 | 5.00E-06 |
| COPD | T2DM | European | rs7236339 | A | G | 0.2277 | 0.0011 | 0.0002 | 1.80E-06 |
| COPD | T2DM | European | rs72736802 | T | A | 0.3485 | 0.0010 | 0.0002 | 1.00E-06 |
| COPD | T2DM | European | rs72763136 | A | G | 0.1996 | 0.0011 | 0.0002 | 4.50E-06 |
| COPD | T2DM | European | rs78395109 | A | G | 0.1704 | 0.0012 | 0.0003 | 2.60E-06 |
| COPD | T2DM | European | rs7942802 | A | G | 0.8562 | -0.0013 | 0.0003 | 4.30E-06 |
| COPD | T2DM | European | rs9319587 | T | C | 0.3919 | -0.0009 | 0.0002 | 4.90E-06 |
| COPD | T2DM | European | rs9508067 | C | A | 0.3841 | -0.0009 | 0.0002 | 3.90E-06 |
| COPD | T2DM | European | rs972113 | T | A | 0.5423 | -0.0009 | 0.0002 | 2.10E-06 |
| COPD | T2DM | East Asian | rs10404911 | T | A | 0.1686 | -0.2016 | 0.0342 | 3.75E-09 |
| COPD | T2DM | East Asian | rs11065983 | A | C | 0.5832 | 0.1566 | 0.0270 | 6.99E-09 |
| COPD | T2DM | East Asian | rs13110699 | G | T | 0.6373 | 0.1495 | 0.0270 | 3.19E-08 |
| COPD | T2DM | East Asian | rs1529672 | A | C | 0.3417 | -0.1531 | 0.0265 | 7.74E-09 |
| COPD | T2DM | East Asian | rs2106696 | A | T | 0.6063 | 0.1701 | 0.0275 | 5.95E-10 |
| COPD | T2DM | East Asian | rs2545769 | A | G | 0.3378 | -0.1649 | 0.0298 | 3.18E-08 |
| COPD | T2DM | East Asian | rs3844442 | A | T | 0.4839 | 0.1591 | 0.0263 | 1.53E-09 |
| COPD | T2DM | East Asian | rs7139170 | C | A | 0.4994 | -0.1419 | 0.0254 | 2.33E-08 |
| COPD | T2DM | East Asian | rs78398471 | A | G | 0.1792 | 0.2297 | 0.0370 | 5.37E-10 |
| COPD | T2DM | East Asian | rs9788721 | T | C | 0.6778 | -0.1802 | 0.0285 | 2.73E-10 |
| T1DM | COPD | European | rs1054000 | C | A | 0.5571 | -0.1590 | 0.0250 | 2.02E-10 |
| T1DM | COPD | European | rs10770140 | T | C | 0.1811 | 0.3190 | 0.0200 | 2.85E-57 |
| T1DM | COPD | European | rs10857988 | A | C | 0.6086 | 0.1900 | 0.0280 | 1.16E-11 |
| T1DM | COPD | European | rs11256448 | G | A | 0.1188 | 0.1420 | 0.0210 | 1.36E-11 |
| T1DM | COPD | European | rs2069408 | G | A | 0.2465 | 0.1470 | 0.0190 | 1.02E-14 |
| T1DM | COPD | European | rs2304256 | A | C | 0.1286 | -0.1390 | 0.0210 | 3.61E-11 |
| T1DM | COPD | European | rs2611215 | G | A | 0.3997 | -0.1680 | 0.0250 | 1.82E-11 |
| T1DM | COPD | European | rs41294605 | A | G | 0.4097 | -0.2240 | 0.0280 | 1.24E-15 |
| T1DM | COPD | European | rs4244808 | G | T | 0.6444 | 0.2280 | 0.0190 | 3.55E-33 |
| T1DM | COPD | European | rs670472 | T | G | 0.3359 | 0.1279 | 0.0190 | 1.62E-11 |
| T1DM | COPD | European | rs6745050 | T | C | 0.6067 | -0.1720 | 0.0190 | 1.40E-19 |
| T1DM | COPD | European | rs7112534 | G | A | 0.6309 | 0.2680 | 0.0330 | 4.62E-16 |
| T2DM | COPD | European | rs10184004 | T | C | 0.4060 | -0.0009 | 0.0002 | 2.20E-07 |
| T2DM | COPD | European | rs12243578 | T | C | 0.2892 | 0.0010 | 0.0002 | 5.10E-08 |
| T2DM | COPD | European | rs7079711 | A | G | 0.1800 | -0.0013 | 0.0002 | 1.80E-08 |
| T2DM | COPD | European | rs72875566 | C | G | 0.1306 | -0.0013 | 0.0002 | 2.70E-07 |
| T2DM | COPD | European | rs9368219 | T | C | 0.1743 | 0.0012 | 0.0002 | 1.30E-07 |
| T2DM | COPD | European | rs9937053 | A | G | 0.4243 | 0.0009 | 0.0002 | 1.30E-07 |
| T2DM | COPD | East Asian | rs10033601 | G | A | 0.4809 | -0.0658 | 0.0070 | 5.46E-21 |
| T2DM | COPD | East Asian | rs10266126 | T | G | 0.2051 | 0.0773 | 0.0082 | 4.23E-21 |
| T2DM | COPD | East Asian | rs10426693 | C | T | 0.2776 | -0.0832 | 0.0079 | 6.17E-26 |
| T2DM | COPD | East Asian | rs10757281 | T | C | 0.2110 | -0.0999 | 0.0088 | 7.23E-30 |
| T2DM | COPD | East Asian | rs10842991 | T | C | 0.3272 | -0.0670 | 0.0073 | 4.39E-20 |
| T2DM | COPD | East Asian | rs10882064 | C | T | 0.5380 | -0.1017 | 0.0075 | 6.91E-42 |
| T2DM | COPD | East Asian | rs1105292 | T | C | 0.5029 | -0.0653 | 0.0070 | 1.07E-20 |
| T2DM | COPD | East Asian | rs11257593 | G | A | 0.4552 | -0.0665 | 0.0068 | 1.38E-22 |
| T2DM | COPD | East Asian | rs11257620 | T | C | 0.2079 | 0.0747 | 0.0081 | 2.91E-20 |
| T2DM | COPD | East Asian | rs11594882 | T | C | 0.2084 | 0.0796 | 0.0083 | 8.78E-22 |
| T2DM | COPD | East Asian | rs11616380 | T | G | 0.2799 | -0.0887 | 0.0076 | 1.79E-31 |
| T2DM | COPD | East Asian | rs116500286 | A | C | 0.0877 | 0.1370 | 0.0128 | 9.84E-27 |
| T2DM | COPD | East Asian | rs116761056 | T | C | 0.0253 | 0.2385 | 0.0222 | 6.38E-27 |
| T2DM | COPD | East Asian | rs11725015 | A | G | 0.3145 | -0.0823 | 0.0079 | 2.06E-25 |
| T2DM | COPD | East Asian | rs12146443 | C | T | 0.6533 | -0.0665 | 0.0071 | 7.52E-21 |
| T2DM | COPD | East Asian | rs1260326 | C | T | 0.4562 | 0.0632 | 0.0066 | 1.01E-21 |
| T2DM | COPD | East Asian | rs12907887 | C | G | 0.2290 | 0.0770 | 0.0083 | 1.74E-20 |
| T2DM | COPD | East Asian | rs13077371 | C | G | 0.4716 | 0.0816 | 0.0071 | 1.43E-30 |
| T2DM | COPD | East Asian | rs13266634 | T | C | 0.4143 | -0.1160 | 0.0067 | 3.72E-67 |
| T2DM | COPD | East Asian | rs1574285 | T | G | 0.5657 | -0.0720 | 0.0069 | 1.72E-25 |
| T2DM | COPD | East Asian | rs163171 | C | T | 0.5865 | 0.1556 | 0.0070 | ###### |
| T2DM | COPD | East Asian | rs16988991 | A | G | 0.4497 | 0.0608 | 0.0066 | 3.20E-20 |
| T2DM | COPD | East Asian | rs17168486 | T | C | 0.4181 | 0.0643 | 0.0067 | 8.23E-22 |
| T2DM | COPD | East Asian | rs1881415 | C | T | 0.5067 | -0.0642 | 0.0067 | 9.51E-22 |
| T2DM | COPD | East Asian | rs2201103 | C | T | 0.6465 | 0.0718 | 0.0072 | 2.02E-23 |
| T2DM | COPD | East Asian | rs231361 | A | G | 0.8180 | 0.0827 | 0.0089 | 1.51E-20 |
| T2DM | COPD | East Asian | rs2796443 | C | T | 0.7104 | -0.0741 | 0.0078 | 2.10E-21 |
| T2DM | COPD | East Asian | rs28584391 | G | T | 0.4929 | 0.0639 | 0.0067 | 1.47E-21 |
| T2DM | COPD | East Asian | rs2977849 | A | T | 0.4952 | 0.0645 | 0.0070 | 3.13E-20 |
| T2DM | COPD | East Asian | rs312457 | A | G | 0.9064 | -0.1085 | 0.0116 | 8.48E-21 |
| T2DM | COPD | East Asian | rs34238147 | A | G | 0.4359 | -0.0640 | 0.0069 | 1.77E-20 |
| T2DM | COPD | East Asian | rs34494123 | T | C | 0.0982 | 0.1241 | 0.0112 | 1.56E-28 |
| T2DM | COPD | East Asian | rs35560038 | T | A | 0.5775 | 0.0660 | 0.0071 | 1.46E-20 |
| T2DM | COPD | East Asian | rs36082564 | A | G | 0.0409 | 0.1835 | 0.0169 | 1.83E-27 |
| T2DM | COPD | East Asian | rs3852527 | G | A | 0.6344 | -0.0959 | 0.0074 | 2.08E-38 |
| T2DM | COPD | East Asian | rs459193 | G | A | 0.4925 | 0.0737 | 0.0066 | 5.94E-29 |
| T2DM | COPD | East Asian | rs4886849 | C | T | 0.4456 | 0.0663 | 0.0071 | 9.81E-21 |
| T2DM | COPD | East Asian | rs60980157 | T | C | 0.0909 | -0.1320 | 0.0135 | 1.40E-22 |
| T2DM | COPD | East Asian | rs62398909 | C | A | 0.4332 | 0.0641 | 0.0069 | 1.54E-20 |
| T2DM | COPD | East Asian | rs6550730 | A | G | 0.1502 | -0.0993 | 0.0096 | 4.47E-25 |
| T2DM | COPD | East Asian | rs6835521 | G | A | 0.5361 | -0.0669 | 0.0072 | 1.52E-20 |
| T2DM | COPD | East Asian | rs7206790 | G | C | 0.1875 | 0.1005 | 0.0093 | 3.21E-27 |
| T2DM | COPD | East Asian | rs72982988 | A | G | 0.1910 | 0.0887 | 0.0088 | 6.80E-24 |
| T2DM | COPD | East Asian | rs73541184 | A | G | 0.0719 | -0.1290 | 0.0138 | 8.95E-21 |
| T2DM | COPD | East Asian | rs7405776 | G | A | 0.6965 | -0.1157 | 0.0080 | 2.09E-47 |
| T2DM | COPD | East Asian | rs742762 | C | A | 0.2882 | -0.0751 | 0.0077 | 1.79E-22 |
| T2DM | COPD | East Asian | rs75307101 | A | T | 0.1594 | -0.0910 | 0.0099 | 3.86E-20 |
| T2DM | COPD | East Asian | rs75933965 | A | G | 0.0137 | 0.3479 | 0.0310 | 3.16E-29 |
| T2DM | COPD | East Asian | rs75974105 | C | G | 0.1332 | 0.1022 | 0.0104 | 8.62E-23 |
| T2DM | COPD | East Asian | rs76393458 | A | G | 0.3054 | 0.1628 | 0.0079 | 2.35E-94 |
| T2DM | COPD | East Asian | rs77883138 | T | C | 0.1497 | 0.0850 | 0.0092 | 2.48E-20 |
| T2DM | COPD | East Asian | rs7947981 | G | A | 0.0987 | -0.1140 | 0.0119 | 9.72E-22 |
| T2DM | COPD | East Asian | rs9465873 | A | G | 0.4366 | 0.0649 | 0.0067 | 3.44E-22 |
| T2DM | COPD | East Asian | rs989099 | G | A | 0.6966 | 0.0662 | 0.0072 | 3.77E-20 |

**Mendelian Randomization Analysis of the Causal Relationship Between Chronic Obstructive Pulmonary Disease and Type 1 and Type 2 Diabetes Mellitus**

Table S2. The corresponding gene names between T1DM and COPD.

| **Exposure** | **Outcome** | **Population** | SNP | Gene name |
| --- | --- | --- | --- | --- |
| T1DM | COPD | European | rs1054000 | MEG3 |
| T1DM | COPD | European | rs10770140 | TH |
| T1DM | COPD | European | rs10857988 | intergenic variant |
| T1DM | COPD | European | rs11256448 | IL2RA |
| T1DM | COPD | European | rs2069408 | CDK2 |
| T1DM | COPD | European | rs2304256 | TYK2 |
| T1DM | COPD | European | rs2611215 | intergenic variant |
| T1DM | COPD | European | rs41294605 | intergenic variant |
| T1DM | COPD | European | rs4244808 | IGF2 |
| T1DM | COPD | European | rs670472 | RNLS |
| T1DM | COPD | European | rs6745050 | intergenic variant |
| T1DM | COPD | European | rs7112534 | intergenic variant |

**Figure S1.** Forward MR analyses of COPD and DM with funnel plot and leave-one-out analysis, European (A, B: COPD-T1DM; D, E: COPD-T2DM), East Asian (E, F: COPD-T2DM).


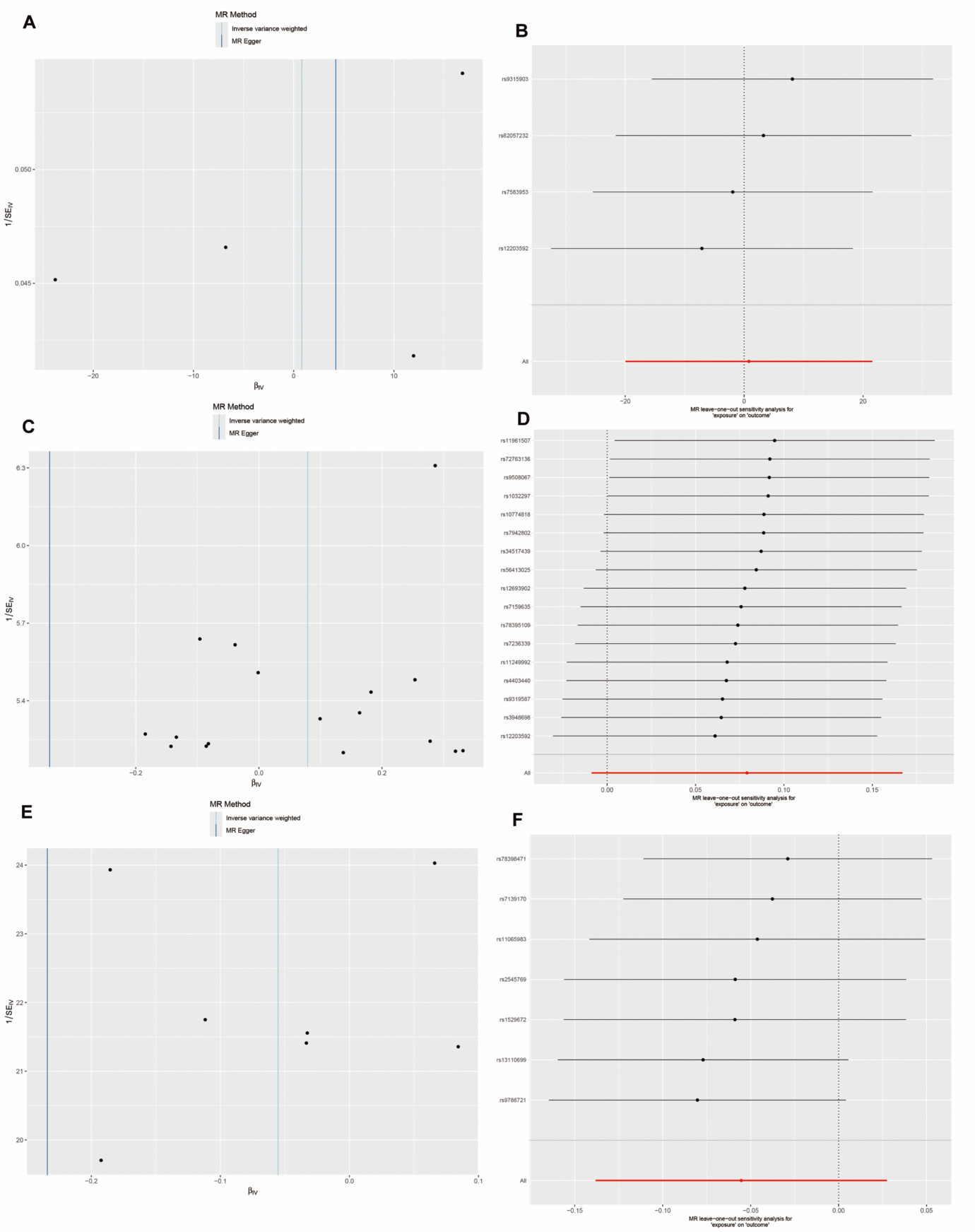


**Figure S2.** Reverse Mendelian randomization analysis of COPD and DM with funnel plot and leave-one-out analysis, European (A, B: T1DM-COPD; D, E: T2DM-COPD), East Asian (E, F: T2DM-COPD).


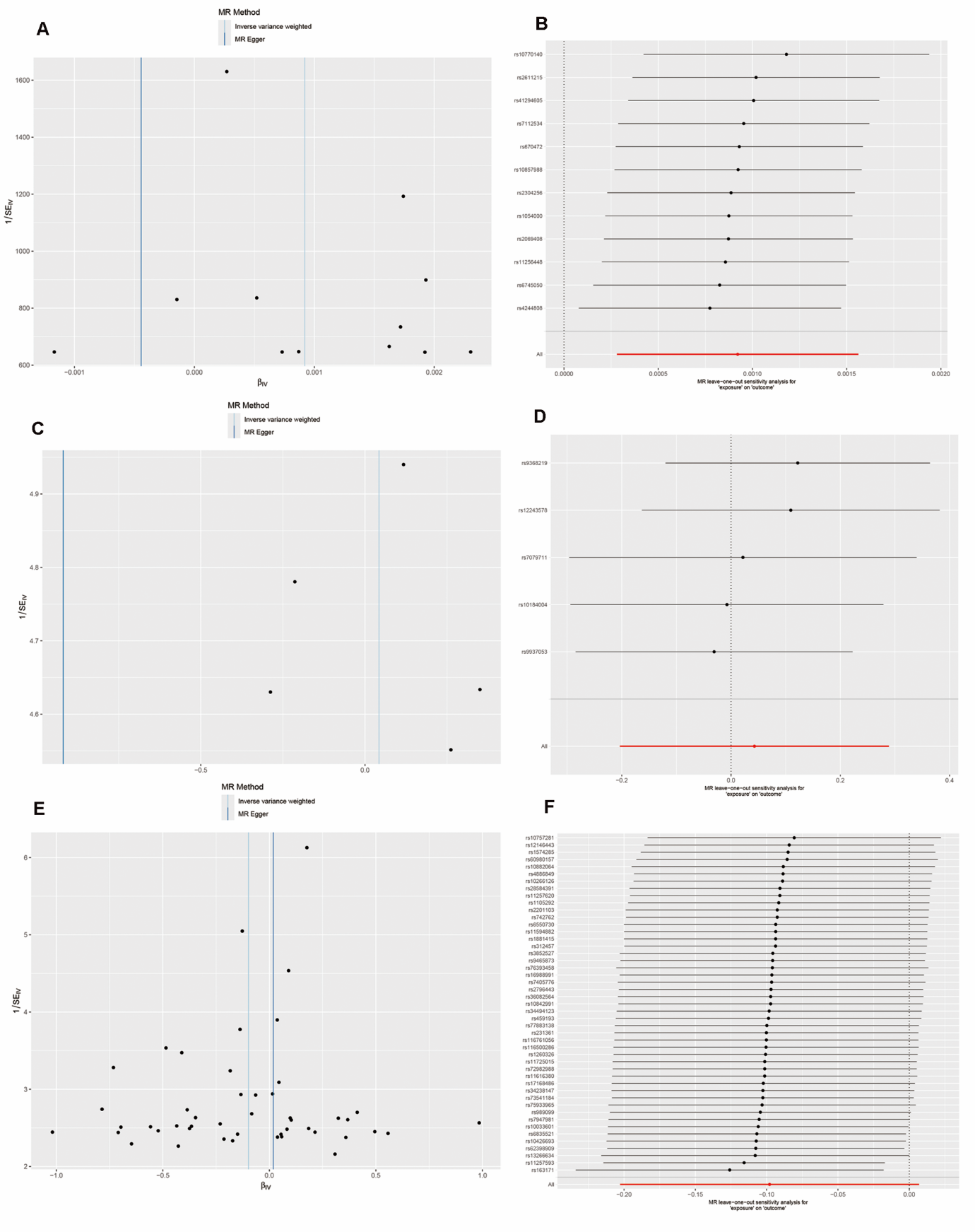

Supplement: Supplementary file 1 [file medi-104-e45935-s001.docx]
